# *Taenia martis* (Cestoda) in captive exotic animals and wild beech marten (*Martes foina*) from the Czech Republic

**DOI:** 10.3389/fvets.2026.1778212

**Published:** 2026-03-13

**Authors:** Ondřej Máca, Roman Vodička, Iva Langrová

**Affiliations:** 1 Department of Zoology and Fisheries, Faculty of Agrobiology, Food and Natural Resources, Czech University of Life Sciences Prague, Prague, Czechia; 2 Department of Pathology and Parasitology, State Veterinary Institute Prague, Prague, Czechia; 3 The Prague Zoological Garden, Prague, Czechia

**Keywords:** Central Europe, common gundi, Cuban hutia, Himalayan striped squirrel, Metacestodes, molecular identity, ring-tailed lemur, zoonotic parasite

## Abstract

**Background:**

Studies focusing on the larval stages of *Taenia martis* remain limited compared with other taeniid species; however, its repeated detection over the past decade highlights its significance for human and animal health, emphasizing the need for further infection-focused research.

**Methods:**

Data from 32 dead animals, including nine common gundis (*Ctenodactylus gundi*), 19 Cuban hutias (*Capromys pilorides*), one Himalayan striped squirrel (*Tamiops mcclellandii*), and three ring-tailed lemurs (*Lemur catta*), which were examined for cysticercosis following necropsy, are presented.

**Results:**

In 10 cases, animals (including four gundis, one squirrel, four hutias, and one lemur) were found positive, with infection intensities ranging from a single larval cyst in the liver to two and 10 metacestodes in the pleural and/or peritoneal cavities. Additionally, one beech marten (*Martes foina*) was found positive for adult cestode, which was molecularly identified as *T*. *martis* for the first time from this definitive host in the Czech Republic.

**Conclusion:**

Molecular tools were employed for accurate species- and haplotype-level identification through characterization of the cytochrome *c* oxidase subunit 1 (*cox1*) mtDNA gene, revealing new and multiple haplotypes, the prevalence of *T. martis* in the studied rodent species, and its first recorded occurrence in *M. foina* in the Czech Republic. Notably, epidemiological and molecular data on these parasites in exotic animals under human care remain limited.

## 1 Introduction

*Taenia martis* (Zeder, 1803) Freeman, 1956 is a widely distributed tapeworm that infects multiple wild vertebrate species across the Northern Hemisphere. Mustelids serve as their primary definitive hosts (DH), while canids, felids, and procyonids are occasionally involved as DH (1–3). The life cycle of *T*. *martis* involves several species of mammals serving as intermediate hosts (IH), where metacestodes are usually found in the pleural and peritoneal cavities. Most records on the identity of DH and IH are based on morphometric and morphological parameters (1, 4).

Over the past two decades, adult *T. martis* have been molecularly confirmed in *Martes martes* (Linnaeus, 1758) and *M. foina* (Erxleben, 1777) from Italy (5), as well as in *Procyon lotor* (Linnaeus, 1758) and *Felis silvestris* Schreber, 1777 from Germany (2, 3). On the other hand, its larval stages were molecularly identified in *Myodes glareolus* (Schreber, 1780) from Croatia, Czech Republic, Denmark, Germany, and Serbia (6–9); *Myodes rufocanus* (Sundevall, 1846) from China and Russia (6); *Apodemus flavicollis* (Melchior, 1834) from Czech Republic, Germany, and Serbia (7–9); *Apodemus sylvaticus* (Linnaeus, 1758) from Serbia and Turkey (6, 9); *Apodemus agrarius* (Pallas, 1771) from Germany (7); *Ondatra zibethicus* (Linnaeus, 1766) from Germany and Luxembourg (10, 11); *Microtus arvalis* (Pallas, 1778) from Czech Republic (8); *Macaca tonkeana* (Meyer, 1899), *Eulemur albifrons* (É. Geoffroy Saint-Hilaire, 1796), *Lemur catta* (Linnaeus, 1758), *Hapalemur alaotrensis* (Rumpler, 1975), and *Callithrix jacchus* (Linnaeus, 1758) from France, Germany, and Italy (12–16), also including reports of human infection from Europe (17–22). As above mentioned, rodents and non-human primates are parasitized by *T*. *martis*, but the examination of other hosts would help to better understand possible transmission pathways or circulation of the parasite.

To date, no reports exist on the occurrence of *T*. *martis* in the Cuban hutia *Capromys pilorides* (Say, 1822), common gundi *Ctenodactylus gundi* (Rothmann, 1776), or the Himalayan striped squirrel (*Tamiops mcclellandii*) (Horsfield, 1840), all of which are kept in captivity. Therefore, this study aims to determine the occurrence and identification of larval *T*. *martis* based on cytochrome *c* oxidase subunit 1 (*cox1*) mtDNA gene and hooks measurements in these exotic rodents, and in the ring-tailed lemur (*L. catta*) from zoological settings. Moreover, to characterize an adult *T*. *martis* from the wild beech marten (*M. foina*) in the Czech Republic.

## 2 Materials and methods

Between 2018 and 2025, nine common gundis (four adult males and five females) from Prague, and Pilsen regions, 19 Cuban hutias (five adult males and seven adult females; five juvenile males and two juvenile females) from Prague, and Ústí nad Labem regions, one Himalayan striped squirrel (adult male) from Liberec region, and three female ring-tailed lemurs from Pilsen region were submitted for necropsy and identification of parasites to the State Veterinary Institute Prague or were necropsied at the Prague Zoological Garden in the Czech Republic. Post-mortem parasitological examinations were conducted, including macroscopic inspection of organs and tissues, followed by microscopic examination, with special emphasis to detect larval stages in the pleural and peritoneal cavities and in liver tissues under light microscopy (LM) using a Leica DM2500 LED optical microscope. Additionally, intestine examination of one wild beech marten (*M. foina*), obtained during routine examination from Pilsen region, revealed the presence of one adult, which was used to study hook measurements and genetic identity. Four metacestodes from three common gundis, 10 from one squirrel, five from three hutias, one from one lemur, and one adult from one marten were washed in physiological saline, fixed in 70% (v/v) ethanol, and stored until molecular analyses were conducted.

DNA was extracted from the 20 metacestodes and a single adult specimen using the NucleoSpin tissue XS kit (Macherey-Nagel, Düren, Germany) according to the instructions of the manufacturer. Polymerase chain reaction (PCR) was performed independently for each specimen. Partial sequences of the *cox1* gene were amplified using the previously published primers JB3 and JB4.5 (23). For each PCR amplification, 5 μL of extracted DNA was used as the template in a 25 μL mixture containing 20 pmol of each primer, GoTaq^®^ G2 Hot Start Green Master Mix (Promega, United States), and nuclease-free water. Thermocycling conditions consisted of an initial denaturation at 94 °C for 3 min, followed by 36 cycles of denaturation at 94 °C for 30 s, annealing at 55 °C for 30 s, extension at 72 °C for 1 min, and a final extension at 72 °C for 10 min. Each PCR product (5 μL) was assessed on a 1.5% agarose gel stained with ethidium bromide and visualized under UV illumination. The products were then purified using the ExoSAP-IT^™^ Express PCR Product Cleanup Reagent Kit (Thermo Fisher Scientific) following the protocol of the manufacturer. Purified amplicons were submitted to Eurofins Genomics (Ebersberg, Germany) for bidirectional Sanger sequencing using the same primers applied in the PCR amplifications. The resulting sequences were compared with those available in the National Center for Biotechnology Information (NCBI) database using the BLASTn algorithm and have been deposited in GeneBank^1^ under the accession numbers (PX765621, PX765622, and PX775690–PX775698).

Multiple sequence alignment was performed using the online MAFFT platform^2^ with standard parameters. Phylogenetic analysis of the *cox1* gene sequences was conducted using the Molecular Evolutionary Genetics Analysis (MEGA) software (version 12.0.11) (24). A phylogenetic tree was generated using the maximum likelihood method based on partial *cox1* sequences (396 bp) and the best-fitting evolutionary model (Tamura–Nei model with a gamma distribution) (25), with bootstrap support calculated from 1,000 replicates.

Hooks were obtained from this adult tapeworm, isolated from *M. foina* and from one metacestode of squirrel, and their measurements were taken following the method described by Lavikainen et al. (26). Morphological parameters, including large and small hook dimensions, were examined using a Nikon SMZ25 microscope.

## 3 Results

**Figure 1:**
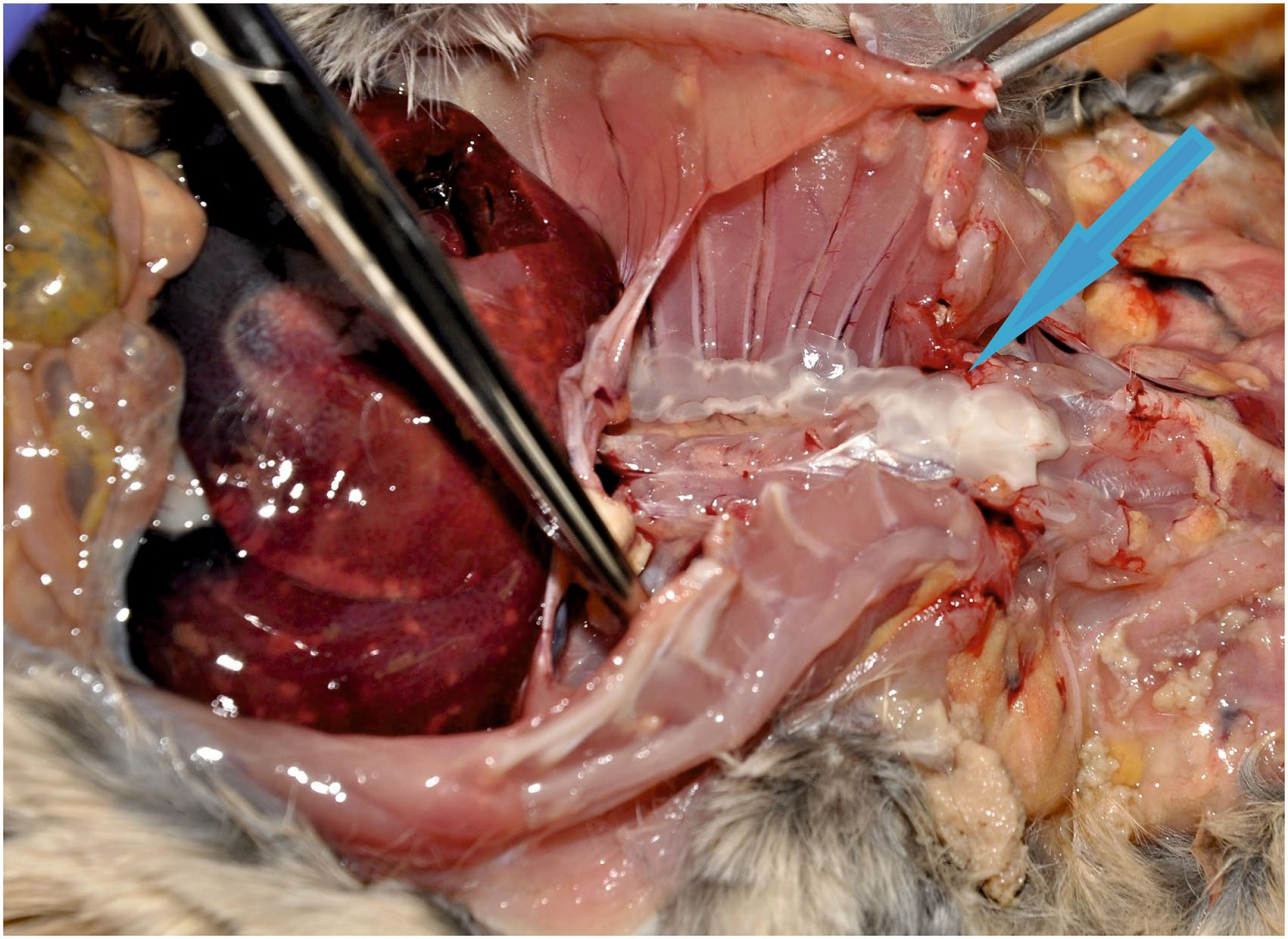
Metacestodes of *Taenia martis* in the pleural cavity of a gundi (blue arrow).

**Figure 2:**
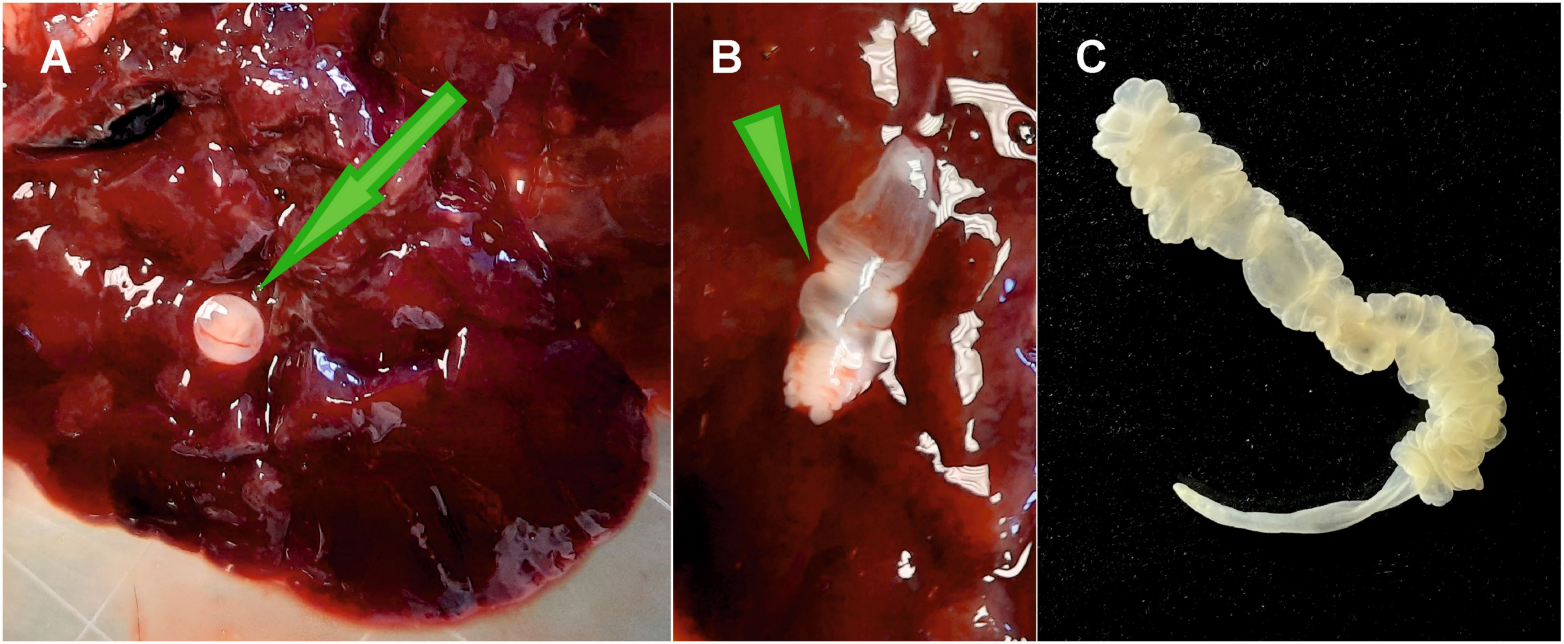
*Taenia martis* in hutia. **(A)** Liver with attached cyst (green arrow). **(B)** Ruptured cysts with released larval stage (green arrowhead). **(C)** Metacestode in the peritoneal cavity showing pseudosegmentation at the anterior region and a narrow, unsegmented end.

Metacestodes were identified as *T*. *martis* in the liver or pleural/peritoneal cavities of 4/9 gundis (two adult males and two adult females; Figure 1) and in 4/19 hutias (two adult females and two males). In hutias, cysts containing metacestodes were located either in the liver (one cyst; Figures 2A,B) or in the peritoneal cavity (one to three metacestodes; Figure 2C). Moreover, 10 metacestodes in one squirrel were localized in the pleural and peritoneal cavities, while one was found in the omentum of a female lemur. One adult *T*. *martis* specimen was found in the small intestine of *M. foina*.

The *cox1* sequences of the present metacestodes (396 bp) from two gundis (GenBank: PX775690 and PX775691), three hutia (PX775692-PX775694), and one lemur (PX775695) were 100% identical to the recently published *T. martis* haplotype TmCZ1 (PQ870824, PQ870825, and PQ870827) detected in wild rodents (*A. flavicollis*, *M. arvalis*, and *M*. *glareolus*) from the Czech Republic, and to haplotype TmaDe1 in *M*. *glareolus* from Denmark (EU544553). Two other hutia isolates (PX765621) and all sequences from one squirrel (PX775697), designated in this study as TmCZ2 haplotype, differed by a single nucleotide and were 99.75% similar to *T*. *martis* haplotype TmCZ1 (PQ870824, PQ870825, and PQ870827), and 100% identical to *T*. *martis* (KJ459910) previously detected in the omentum of *L. catta* from an Italian zoological garden. Both haplotypes (TmCZ1 and TmCZ2) were present in one hutia. Two sequences from another gundi differed from each other and were designated as haplotype TmCZ2 (PX775696) and a novel haplotype, TmCZ3 (PX765622), exhibiting 99.75% similarity to TmCZ1. The adult *T*. *martis* obtained from the intestine of *Martes foina* (PX775698) was 99.75% identical to TmCZ1, 99.49% TmCZ2, and 100% to TmCZ3.

**Figure 3:**
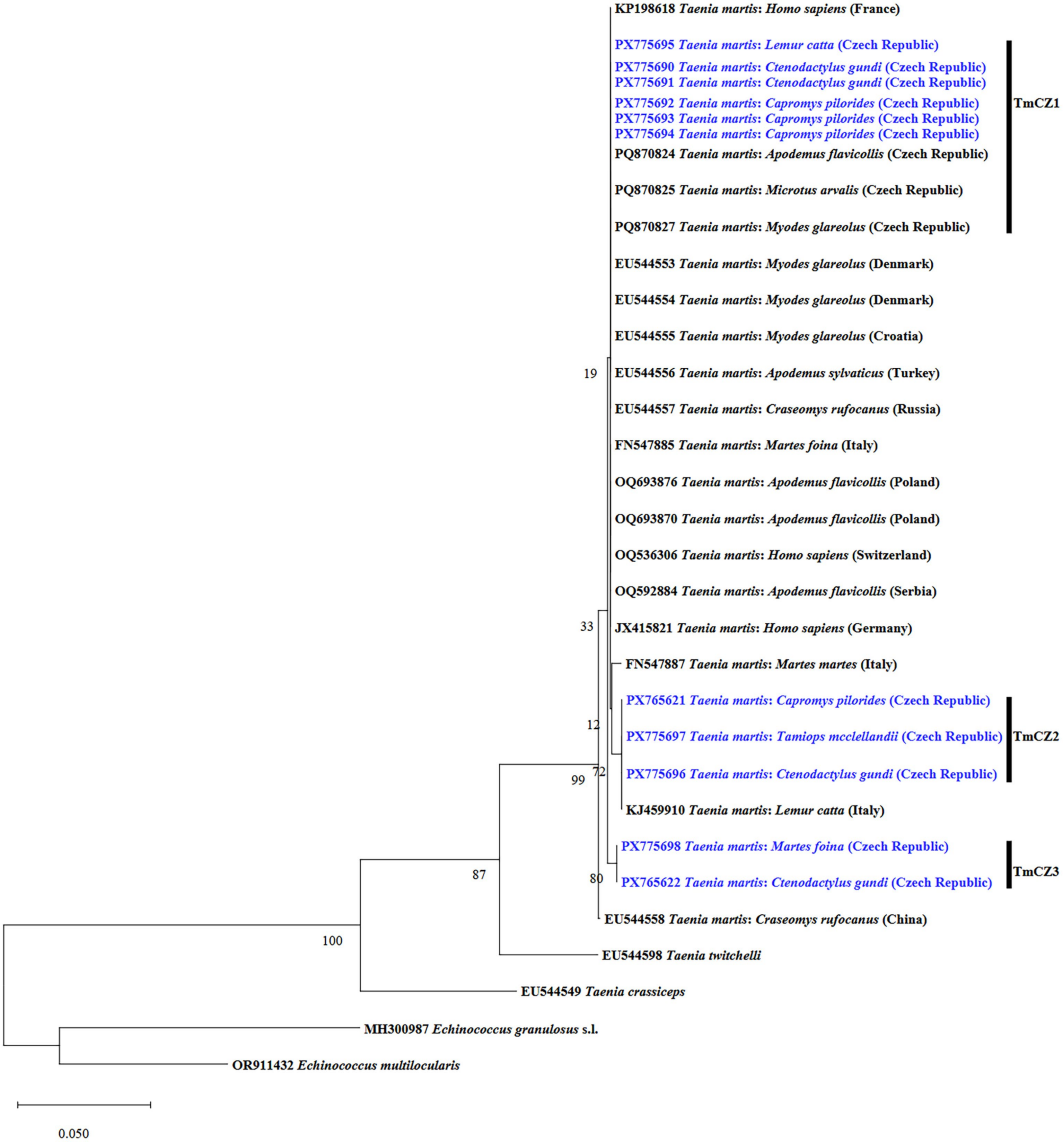
Phylogenetic relationships of *Taenia martis* isolates from the present study (bolt and blue) relative to other sequenced members of the genus *Taenia* (previously published). The tree was inferred using the maximum likelihood method based on partial cytochrome *c* oxidase subunit 1 (*cox1*) mtDNA marker sequences, applying the Tamura–Nei model with gamma distribution and 1,000 bootstrap replicates. The new *T. martis* isolates in this study are indicated in bold and blue.

Phylogenetic analyses confirmed the identity of *T*. *martis* and other haplotypes in comparison with previously described isolates of this parasite species from different regions and hosts (Figure 3). Specifically, the results showed that all *T*. *martis* isolates identified in this study (TmCZ1, TmCZ2, and TmCZ3) were grouped into three well-supported clusters.

**Figure 4:**
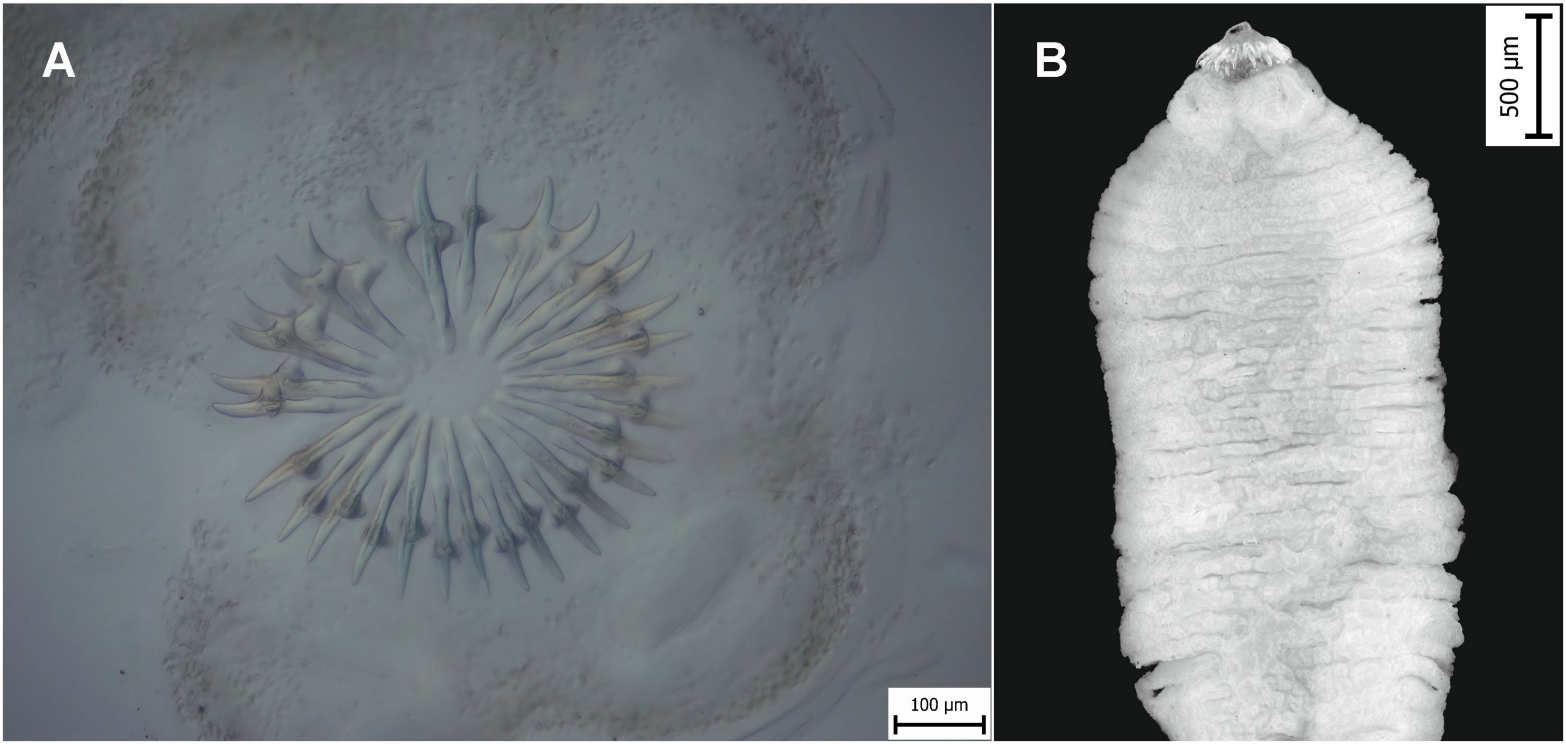
Photomicrographs of *Taenia martis*. **(A)** Rostellum with 30 hooks in a metacestode from the Himalayan striped squirrel. **(B)** Adult tapeworm from beech marten.

Thirty well-developed rostellar hooks were obtained from one metacestode found in the Himalayan striped squirrel (Figure 4A), of which 15 large hooks measured 203–212 μm long and 15 small ones measured 155–182 μm long (Table 1). The adult tapeworm from *M. foina* contained 12 hooks in each row (Figure 4B) with differing dimensions (Table 2).

**Table 1:** Large and small rostellar hook dimensions of adult *Taenia martis* from *Tamiops mcclellandii.*

Character		Large hooks (μm)			Small hooks (μm)		
	*N*	Min	Max	Mean/SD	Min	Max	Mean/SD
Total length	5	203	212	208/3.7	155	182	166.2/11.6
Total width	5	78	82	81.2/1.9	55	69	59.4/9.6
Basal length	5	126	136	130.2/4	105	116	111.2/4.3
Apical length	5	92	95	93.6/1.3	67	79	72.4/5
Guard length	5	32	36	34.4/1.7	29	31	30.2/0.8
Guard width	5	23	30	28/2.9	23	29	24/3.6
Blade curvature	5	22	27	23/2	12	22	17.6/5.6
Handle width	5	24	41	34.4/6.9	19	21	18.4/2.6

**Table 2:** Large and small rostellar hook dimensions of adult *Taenia martis* from *Martes foina.*

Character		Large hooks (μm)			Small hooks (μm)		
	*N*	Min	Max	Mean/SD	Min	Max	Mean/SD
Total length	5	219	222	220/1.1	168.6	175.5	172.3/2.8
Total width	5	90.9	94.2	92.9/1.3	79.2	81.3	80.3/0.8
Basal length	5	140.9	143.7	142.8/1.1	120.1	124.5	121.8/1.6
Apical length	5	106.1	107.9	107/0.7	75	77.8	76.7/1.1
Guard length	5	41.7	48.2	44.9/2.9	30.4	38.5	35.2/4
Guard width	5	28.4	31.6	30.1/1.2	19.5	23.6	22.6/1.7
Blade curvature	5	21.4	30.2	26.1/3.4	19.1	22.7	20.1/1.5
Handle width	5	32.7	37.7	35.3/1.8	15.4	17.9	16.5/1.1

## 4 Discussion

The present findings represent new host and geographical records of metacestodes, whose identification was based on the metacestode shape and hooks measurements and supported by molecular analyses that confirmed their identity as *T*. *martis*. Even though this cestode species has been recently reported worldwide in exotic hosts, including non-human and human primates (14, 15, 22, 27), the identity of IH and DH of *T*. *martis* by molecular analyses is still fragmentary. Therefore, the present genetic analysis increases the knowledge on these larval stages.

Our understanding on the occurrence of larval stages (metacestodes) of several taeniid species, including *T*. *martis*, in wild rodents across the Czech Republic has significantly improved in recent years (8). However, data on the genetic identity of *T. martis* from captive IHs and DHs remain unknown in our country. Therefore, this study enriches the national epidemiological dataset by identifying previously undescribed *T*. *martis* haplotype (TmCz2) in Czech Republic, and a novel haplotype (TmCz3) not previously reported. Of particular interest, for the first time, multiple *T*. *martis* haplotypes were observed within a single individual, indicating that all metacestodes should be individually characterized, as published for other taeniids (8, 11, 28, 29), when identification extends beyond routine diagnostics. These findings could raise new questions, including the source of infection, transmission pathways, and the occurrence of multiple haplotypes in wildlife DHs for upcoming research.

The shape, number, and morphometrics of the rostellar hooks in metacestode from the Himalayan striped squirrel (large hooks 203–212 μm; small hooks 155–182 μm) fall within the ranges reported for *T*. *martis* (195–250 μm and 156–214 μm, respectively) by Prokopič (30) and Loos-Frank et al. (2, 4). Other tapeworm species whose metacestodes develop in rodent IH exhibit overlapping hook size ranges, including medium-sized hooks of *Taenia crassiceps* (146–209 μm; 114–156 μm) and *Taenia pisiformis* (200–300 μm; 114–177 μm) (31–33). Larvae of *T. pisiformis* display a characteristic shape and a large number of hooks (34–46) (31). Morphological parameters of *T*. *martis* have served as the only identification method in the past (31–34), although the specimens examined in this study are not clearly assignable to *T*. *martis* based on the hook sizes, thus indicating that molecular identification is the most reliable approach for the species determination, and only way when metacestodes are without hooks [see Husák et al. (8)], fragmented or autolytic.

The occurrence of parasitic infections in zoological facilities should be monitored, since soil or water might be contaminated with tapeworm eggs and thus constitute a potential source of infection (35, 36). Additionally, the proximity of wildlife and domestic or stray animals (e.g., Mustelidae, Canidae, Felidae, and Procyonidae) (2, 3, 5) to the zoological enclosures (RV, personal observation) might potentially increase the risk of transmission as occurred with *M. foina* in this study. Effective control of parasitic infections in exotic DH animals kept in zoological gardens requires appropriate parasitological testing, including fecal examination, followed by molecular characterization, as well as the detection and tracing of potential external/internal sources that might contribute to the infection.

Similarly, *T. martis* possess a potential risk to other accidental hosts, as zoo animals (e.g., *L. catta*, *M. tonkeana*, *E. albifrons*, *C. jacchus*, and *H. alaotrensis*) (12–16). In these hosts, infection might depend on the immune status, lead to severe disease or even death (1), a risk that cannot be excluded for the species examined in the present study. In recent years, *T*. *martis* metacestodes have been detected in humans, often associated with exposure during gardening activities (17–19, 21, 22, 27). Hypothetically, the consumption of unwashed fruits or vegetables represents a possible source of infection for humans and zoo animals, as noted by Mueller et al. (21).

In this study, we report a new exotic IH and geographical record (Czech Republic) for *T*. *martis*, alongside the first integrative taxonomic analysis of this cestode, combining hook morphometrics and molecular characterization.

## Data Availability

The datasets presented in this study can be found in online repositories. The names of the repository/repositories and accession number(s) can be found in the article/supplementary material.

## Footnotes

1
http://www.ncbi.nlm.nih.gov/pubmed

2
https://mafft.cbrc.jp/alignment/server/
